# MoS_2_ Nanosheet–Pd Nanoparticle Composite for Highly Sensitive Room Temperature Detection of Hydrogen

**DOI:** 10.1002/advs.201500004

**Published:** 2015-03-02

**Authors:** Cihan Kuru, Chulmin Choi, Alireza Kargar, Duyoung Choi, Young Jin Kim, Chin Hung Liu, Serdar Yavuz, Sungho Jin

**Affiliations:** ^1^ Materials Science and Engineering ProgramUniversity of California‐San DiegoLa JollaCA92093USA; ^2^ Department of Mechanical and Aerospace EngineeringUniversity of California‐San DiegoLa JollaCA92093USA; ^3^ Department of Electrical and Computer EngineeringUniversity of California‐San DiegoLa JollaCA92093USA

**Keywords:** hydrogen sensing, MoS_2_ nanosheet, room temperature, solvent exfoliation

## Abstract

**Highly sensitive hydrogen detection at room temperature** can be realized by employing solution‐processed MoS_2_ nanosheet–Pd nanoparticle composite. A MoS_2_–Pd composite exhibits greater sensing performance than its graphene counterpart, indicating that solvent exfoliated MoS_2_ holds great promise for inexpensive and scalable fabrication of highly sensitive chemical sensors.

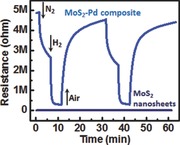

Hydrogen, a clean and abundant energy source, has been utilized in fuel cells to generate electricity with the aim of reducing the dependence on fossil fuels. However, hydrogen is a colorless, odorless, tasteless, flammable, and explosive gas, which arises some safety concerns. For the safe implementation of fuel cells, hydrogen leaks have to be detected before hydrogen concentration reaches a hazardous level.^1^, ^2^ Metal oxide sensors^3^, ^4^, ^5^, ^6^ are effective for the detection of hydrogen; however they require high operation temperature, which increases the power consumption as well as posing a risk for safety itself since hydrogen is highly flammable at elevated temperatures.^7^, ^8^ In this regard, developing reliable hydrogen detection technologies which can operate at room temperature is highly desirable.

2D materials have drawn tremendous attention in recent years due to their novel and unique electronic, optical, and mechanical properties.^9^, ^10^, ^11^ Moreover, their high surface‐to‐volume ratio makes them attractive for sensing applications. Graphene, a 2D material made of carbon, has been shown to be an effective sensing platform for toxic gases such as NO_2_ and NH_3_.^12^, ^13^, ^14^, ^15^ Decorating graphene with metal nanoparticles (NPs) such as Pt, Pd, Au, or Ag increases the sensor response due to their catalytic effect.^16^, ^17^ Moreover, Pd‐decorated graphene has been demonstrated as a hydrogen sensor,^18^, ^19^ in which modulation of Pd work function causes a change in the amount of net doping in graphene leading to a resistance change showing a response to hydrogen.

Recently, molybdenum disulfide (MoS_2_) has been explored for electronic applications due to its sizable band gap (1.2 and 1.8 eV for bulk and single layer, respectively), which enables its conductivity to be modulated by a gate voltage.^20^, ^21^ Similar to graphene, MoS_2_ has a layered structure, where each layer consists of covalently bonded Mo–S atoms and the neighboring layers attach each other by van der Waals forces.^22^ MoS_2_ can be obtained by mechanical or chemical exfoliation of bulk MoS_2_ or can be grown by Chemical Vapor Deposition (CVD).^23^, ^24^, ^25^ Perkins *et al*. have demonstrated mechanically exfoliated single layer MoS_2_ flake as a chemical sensor, in which monolayer MoS_2_ shows a strong response to electron donors (triethylamine) and a lower response to electron acceptors (acetone) with detection limits of 10 ppb (parts per billion) and 500 ppm (parts per million), respectively, attributed to the n‐type nature of MoS_2_.^26^ However, mechanical exfoliation is a low yield method and is not suitable for practical applications. On the other hand, CVD grown MoS_2_ films have also been investigated for the gas sensing and a strong response was found towards NH_3_ with a detection limit of 300 ppb.^27^ Although CVD method seems to provide a solution for the scalable growth of MoS_2_, high temperature growth conditions (750–1000 °C)^24^, ^27^ pose a barrier for inexpensive fabrication of chemical sensors.

Chemical exfoliation of MoS_2_ is favorable for the large scale and low cost production of MoS_2_ chemical sensors. A lithium intercalation method^28^ can be used to exfoliate bulk MoS_2_ crystals to produce single layer MoS_2_ nanosheets. However, this method requires a long lithiation process (3 days) and results in MoS_2_ nanosheets with traces of lithium particles, which degrades the MoS_2_ semiconducting properties.^29^ On the other hand, solvent exfoliation method^30^ can provide high yield and fast production of a few layer MoS_2_ nanosheets, in which exfoliation takes place by ultrasonication of bulk MoS_2_ in suitable solvents such as *N*‐methyl‐pyrrolidone (NMP) or isopropanol whose surface tension is in the range of 30–40 mJ m^−2^, which facilitates the exfoliation process.^31^, ^32^

In this work, we present solution‐processed MoS_2_ nanosheet–Pd nanoparticle composite for H_2_ sensing at room temperature, in which MoS_2_–Pd composite show remarkable electrical response towards H_2_ with excellent response and recovery times. A few‐layers MoS_2_ nanosheets can be produced by a facile solvent exfoliation method and the MoS_2_–Pd composite can be fabricated by simply drop casting of MoS_2_–PdCl_2_ solution and subsequent annealing process. The effect of annealing time on H_2_ sensing performance of MoS_2_–Pd composite is investigated. The sensing mechanism is studied by transport measurements of MoS_2_ nanosheets and MoS_2_–Pd composite by fabricating field effect transistor (FET) devices. We also compare the H_2_ sensing performance of MoS_2_–Pd composite with graphene–Pd composite, fabricated in a similar fashion, revealing that MoS_2_–Pd exhibits much higher sensor response with shorter response and recovery times and indicating that 2D MoS_2_ is a promising candidate for highly sensitive room temperature gas detection.

MoS_2_–Pd composite was prepared by drop casting of MoS_2_–PdCl_2_ solution (**Figure** **1**a) on SiO_2_‐coated Si substrates with subsequent annealing process to reduce PdCl_2_ (see the Experimental Section for details). The optical image of sensor device is shown in Figure 1b. Figure 1c,d shows tilted‐view scanning electron microscopy (SEM) images of the MoS_2_–Pd composite film, in which MoS_2_–Pd composite forms a continuous film (around 500 nm thick) in a self‐assembled manner. From the top‐view SEM image (Figure 1e), it is clear that MoS_2_ nanosheets are highly exfoliated as they appear transparent. In order to further understand the layered structure of MoS_2_, AFM (Atomic Force Microscopy) measurements and thickness analysis of the MoS_2_ nanosheets dispersed on a Si substrate were carried out. We measured the thickness of nine MoS_2_ nanosheets, in which we found that the thickness of the nanosheets range from 2.2 to 25.8 nm, with the majority of them having a thickness less than 10 nm. By considering the thickness of the single layer MoS_2_ being 0.65 nm, the number of layers is estimated to range from 3 to 40 (Figure S1, Supporting Information). Figure 1f illustrates the schematic diagram of the MoS_2_–Pd composite, in which Pd NPs (20–100 nm diameter) are sandwiched by MoS_2_ nanosheets.

**Figure 1:**
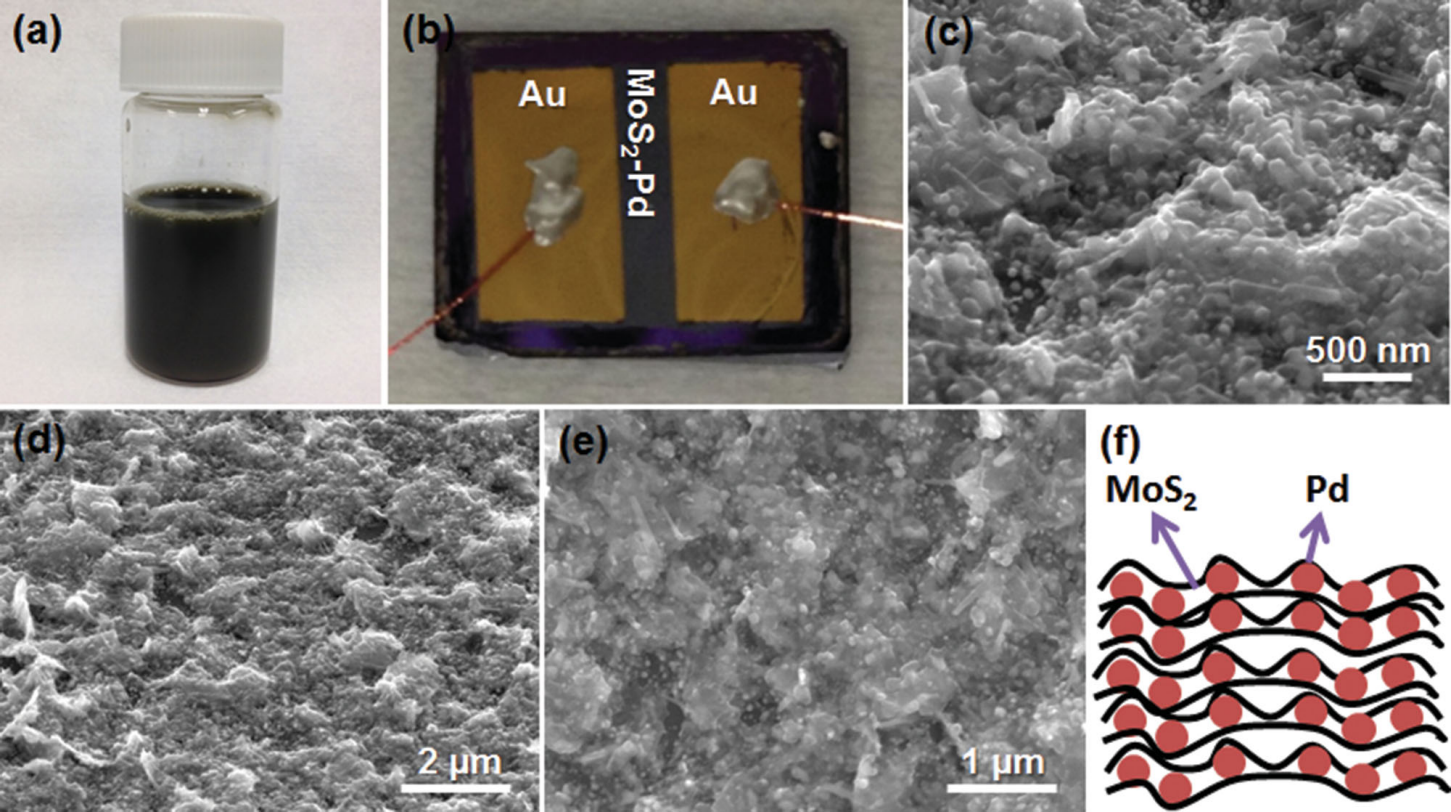
Optical image of a) MoS_2_–PdCl_2_ solution and b) MoS_2_–Pd composite sensor device. c) High and d) low magnification tilted‐view, and e) top‐view SEM images of MoS_2_–Pd composite. f) Schematic illustration of MoS_2_–Pd composite.

X‐ray diffraction (XRD) analyses (**Figures** **2**a and S2, Supporting Information) were carried out in order to evaluate the crystal structure of bulk MoS_2_ powder, MoS_2_ nanosheets, and MoS_2_–Pd composite. XRD pattern of bulk MoS_2_ powder shows the main peaks of molybdenite‐2H, in which a strong peak is observed at 2*θ* ≈ 14.4^o^ (002), indicating that MoS_2_ powder is highly crystalline.^33^ On the other hand, MoS_2_ nanosheets and MoS_2_–Pd composite also showed the (002) peaks with smaller intensities, indicating that MoS_2_ is highly exfoliated after ultrasonication.^34^, ^35^ After exfoliation, the position of the (002) peak slightly shifted to lower angle due to the increased interlayer spacing. Furthermore, we observed that the intensity of the (002) peak is the smallest for MoS_2_–Pd composite, which can be attributed to the possibility that MoS_2_ nanosheets are precluded from restacking by Pd NPs. To further analyze MoS_2_, Raman spectroscopy measurements were performed (Figure 2b), in which the characteristic Raman shifts of MoS_2_ (E^1^_2g_ and A_1g_)^36^ were observed for all the samples.

**Figure 2:**
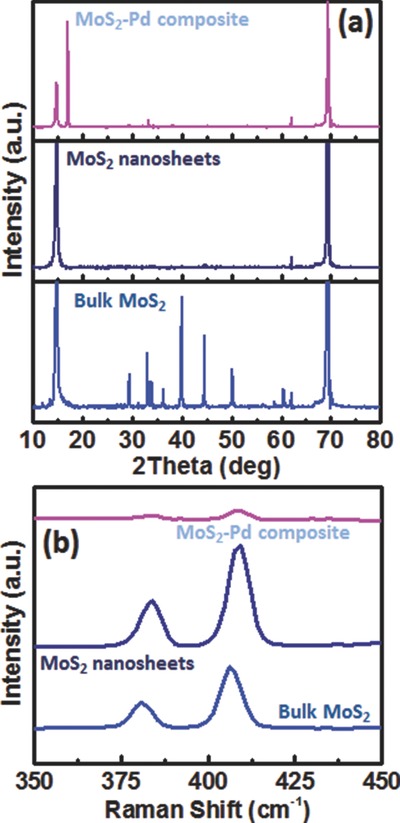
a) XRD patterns and b) Raman spectra of bulk MoS_2_, MoS_2_ nanosheets, and MoS_2_–Pd composite.

Electrical response of the sensors to H_2_ was evaluated by flowing H_2_ in N_2_ with 200 sccm (standard cubic centimeters per minute) flow rate at room temperature. In our measurements, the effect of N_2_ on sensor response is strictly eliminated by flowing N_2_ prior to H_2_ until the sensor response is stabilized. The MoS_2_ nanosheets and MoS_2_–Pd composite show a strong response to N_2_, which could be explained by the fact that O_2_ molecules are pushed outside of the chamber by N_2_ flow, in which p‐doping effect of O_2_ vanishes. O_2_ adsorption is known to lead significant hole doping in graphene,^37^ hence a similar effect can be expected for MoS_2_. The sensor response is defined as *R*_1_/*R*_2_, where *R*_1_ and *R*_2_ are the resistance of the sensor device in N_2_ and H_2_, respectively. **Figure** **3**a shows the electrical response of MoS_2_ nanosheets and MoS_2_–Pd composite to 50 000 ppm of H_2_, in which MoS_2_ nanosheets do not show any significant response to H_2_ exposure (see Figure S3, Supporting Information for the zoomed‐in plot to see the details of response) while MoS_2_–Pd composite shows a strong response. Pd NPs serve as the sensing material, where the work function of Pd shifts upon H_2_ exposure due to the formation of PdH*_x_* compounds.^38^ H_2_ molecules can dissociate on the surface of Pd and diffuse into the Pd lattice changing its work function.^39^ As a result, the doping amount in MoS_2_ is altered by changing the overall resistance of the device. The role of 2D MoS_2_ is crucial since it serves as a platform for the attachment of the Pd NPs and provides high surface‐to‐volume‐ratio and low charge carrier density in the background due to its semiconducting nature, which makes it highly sensitive to H_2_ exposure. The resistance of MoS_2_–Pd composite device exhibits a sharp decrease with H_2_ exposure followed by saturation, with the sensor response being about 10, as well as the sensor shows complete recovery in air without any heating or UV irradiation. Desorption of hydrogen atoms from Pd takes place in the presence of O_2_ by forming H_2_O,^40^ which in turn recovers the sensor. The sensor response and recovery times are defined as the amount of the time required for the sensor resistance to reach 90% of its saturation and to recover to 90% of its ground state, respectively.^41^ MoS_2_–Pd composite sensor device has response and recovery times of 40 and 83 s, respectively. *I*–*V* measurements were performed before and after H_2_ exposure (Figure 3b), in which linear *I*–*V* responses are obtained. This ensures that no Schottky barrier forms between Ti/Au contacts and MoS_2_–Pd composite and the channel itself is responsible for the resistance change upon H_2_ exposure rather than the modulation of Schottky barrier height. We also performed sensing measurements at different concentrations of H_2_, ranging from 50 000 to 500 ppm by 40 s pulses (Figure 3c). Figure 3d shows the recovery time and sensor response as a function of H_2_ concentration, in which both the recovery time and sensor response decrease with the decreasing H_2_ concentration. As the partial pressure of H_2_ is decreased, the amount of hydrogen uptake into the Pd NPs is reduced resulting in a lower sensor response. The sensor response exhibits almost a linear trend for concentrations of 500–25 000 ppm and tends to deviate to a saturation trend at higher concentrations.

**Figure 3:**
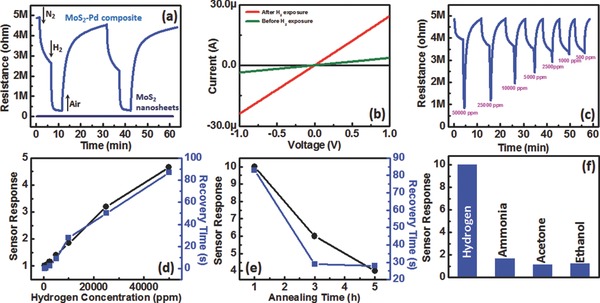
a) Electrical responses of pristine MoS_2_ nanosheets and MoS_2_–Pd composite to 50 000 ppm H_2_. b) *I*–*V* characteristics of MoS_2_–Pd composite before and after H_2_ exposure. c) Electrical response of MoS_2_–Pd composite exposed to different concentrations of H_2_ (500–50 000 ppm) by 40 s pulses. d) Recovery time and sensor response of MoS_2_–Pd composite as a function of H_2_ concentration. e) Recovery time and sensor response of MoS_2_–Pd composite as a function of annealing time. f) Cross‐sensitivity of MoS_2_–Pd composite to 50 000 ppm hydrogen, 50 ppm ammonia, 50 000 ppm acetone and ethanol.

The effect of annealing time on the sensor characteristics of MoS_2_–Pd composite was investigated. Figure 3e shows the recovery time and sensor response of the MoS_2_–Pd composite sensors annealed for various time durations. Increasing the annealing time significantly improves the recovery of the sensor at the expense of reduced sensor response. For example, the recovery times/sensor responses are 83 s/10, 29 s/6, and 28 s/4 for 1, 3, and 5 h annealed samples, respectively. SEM analysis (Figure S4, Supporting Information) of the samples show that annealing changes the morphology of the film into a more spaced structure, which helps the recovery of the sensors. On the other hand, reduced sensor response could be explained by the fact that annealing turns MoS_2_ nanosheets into a more agglomerated structure resulting in a decrease in the number of Pd NPs which contact to MoS_2_ nanosheets.

We also investigated the cross‐sensitivity of MoS_2_–Pd composite to ammonia, ethanol and acetone. As shown in Figure 3f, the sensor exhibits a sensor response of 10, 1.65, 1.13, and 1.22 to 50 000 ppm hydrogen, 50 ppm ammonia, 50 000 ppm acetone and ethanol, respectively, indicating that MoS_2_–Pd composite has a little cross‐sensitivity to these gases.

As a comparison, we fabricated graphene–Pd composite sensor and measured its electrical response to 50 000 ppm of H_2_ (Figure S5, Supporting Information), in which graphene–Pd composite (Figure S6, Supporting Information) shows a sensor response of only 1.34 with a response time of 102 s and incomplete recovery in 30 min. Unlike MoS_2_–Pd, the resistance of graphene–Pd composite increases with H_2_ exposure indicating that graphene–Pd composite are initially p‐doped and the reduction in the work function of Pd upon H_2_ exposure leads to partial depletion of holes in graphene increasing its resistance. It is clearly seen that MoS_2_–Pd composite exhibits superior H_2_ sensing performance than its graphene counterpart indicating that 2D MoS_2_ is more promising for room temperature hydrogen detection.

In order to elucidate the sensing mechanism of MoS_2_–Pd composite, transport measurements were carried out by fabricating FET devices. **Figure** **4** shows the transport data of MoS_2_ nanosheets and MoS_2_–Pd composite, in which MoS_2_ nanosheets and MoS_2_–Pd composite both show n‐type transport behavior with a large shift to the positive side in threshold voltage for MoS_2_–Pd composite. This indicates Pd NPs have a p‐doping effect on MoS_2_ causing partial depletion of electrons. Based on these results, we believe that work function of Pd is higher than that of MoS_2_ before H_2_ exposure, which is consistent with the reported work function values of Pd (5.1–5.6 eV)^42^, ^43^ and MoS_2_ (4.3–5.2 eV).^44^, ^45^ After H_2_ exposure, work function of Pd decreases significantly resulting in a recovery of depleted electrons in MoS_2_, which in turn reduces the overall resistance.

**Figure 4:**
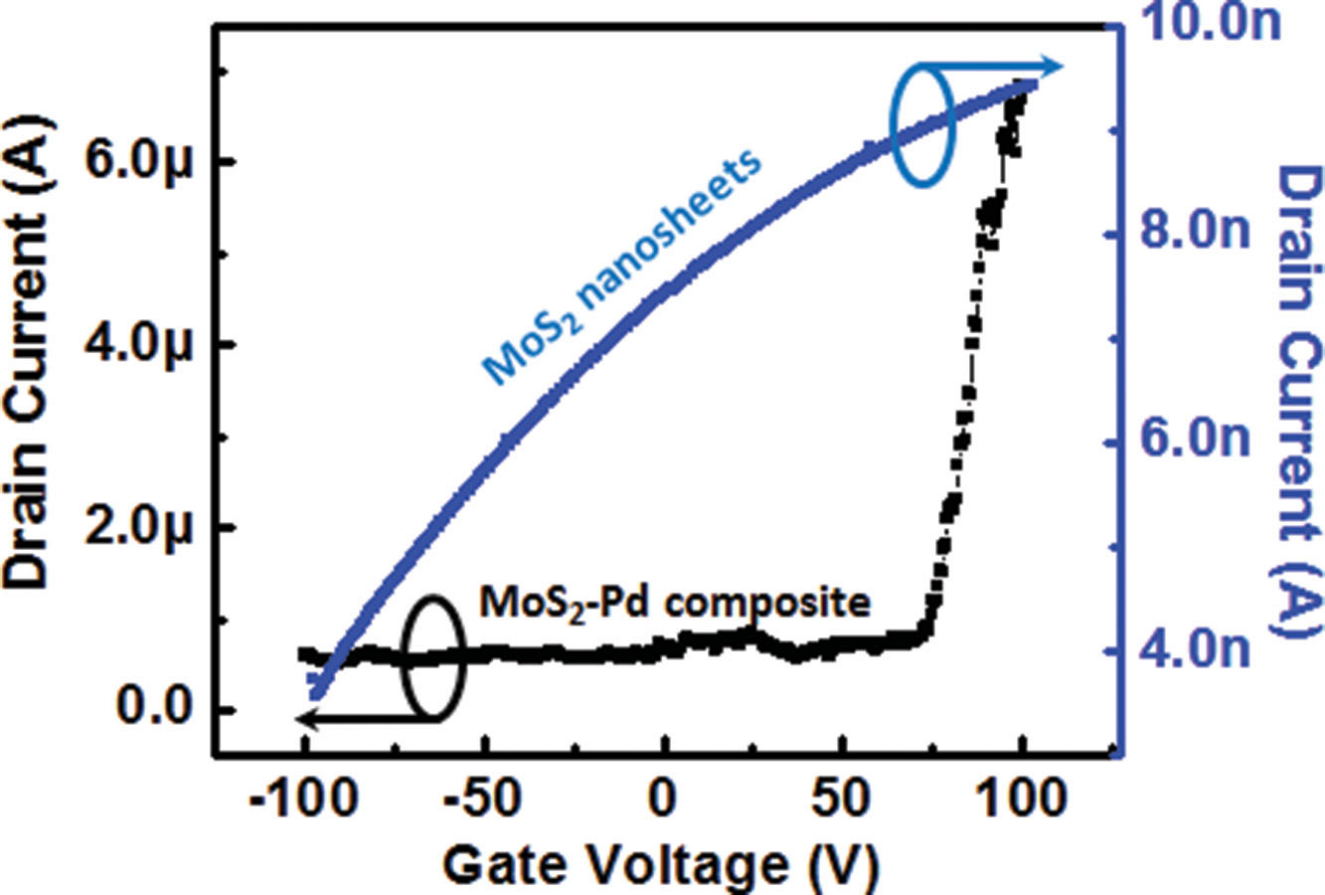
Drain current versus gate voltage of MoS_2_ nanosheets and MoS_2_–Pd composite.

In summary, we demonstrated highly sensitive detection of H_2_ at room temperature by employing solution‐processed MoS_2_ nanosheet–Pd nanoparticle composite, which can be readily fabricated by a facile solvent exfoliation and drop casting method. In particular, MoS_2_–Pd composite sensor exhibits a sensor response of around 10 toward 50 000 ppm H_2_ with a response and recovery time of 40 and 83 s, respectively. Pd NPs enable sensitivity toward H_2_ based on work function modulation of Pd providing high sensitivity, fast response, and recovery. Recovery time can be further decreased down to 28 s by increasing the annealing time. Furthermore, the sensing performance of MoS_2_–Pd was compared with graphene–Pd composite film, in which MoS_2_–Pd outperforms graphene–Pd composite film. These results indicate that chemically exfoliated MoS_2_ holds a great potential for the inexpensive and scalable fabrication of high sensitivity chemical sensors.

## Experimental Section

*Materials*: Bulk MoS_2_ powder (5 μm powder size), NMP, and palladium chloride (PdCl_2_) were purchased from Sigma Aldrich. Bulk graphite flakes were purchased from Graphene Supermarket.

*Preparation of MoS_2_–PdCl_2_ and Graphene–PdCl_2_ Solutions*: A 400 mg bulk MoS_2_ powder was mixed with 80 mL NMP and then the mixture was probe sonicated (750 W and 80% amplitude) in an ice bath for 2 h to exfoliate bulk MoS_2_. The resultant solution was then centrifuged at 1500 rpm for 45 min to remove any remaining bulk particles. After that, NMP was evaporated in a vacuum oven followed by redispersion of MoS_2_ nanosheets in deionized water with a concentration of 1.5 mg mL^−1^. MoS_2_–PdCl_2_ solution was prepared by adding 30 mg PdCl_2_ into 20 mL of MoS_2_–water solution and a subsequent sonication for 30 min. Graphene–PdCl_2_ solution was prepared by following the same procedure.

*Fabrication of Hydrogen Sensors*: A 0.5 mL of prepared MoS_2_–PdCl_2_ and graphene‐PdCl_2_ solution was dropped on SiO_2_‐coated Si substrates, followed by baking on a hot plate at 100 °C until the solution is dried. The resultant film was then annealed in forming gas atmosphere at 400 °C to reduce PdCl_2_ and remove any remaining NMP. In order to fabricate the contacts for sensing measurements a piece of Teflon tape was used as a mask to define the channel (2 mm channel length and 1 cm width) and subsequent sputter deposition of Ti/Au (10/150 nm) was performed. For the fabrication of MoS_2_ nanosheet sensors, MoS_2_ nanosheets which were dispersed in ethanol was spin coated on SiO_2_‐coated Si substrate and then photolithography and following deposition of Ti/Au (10/150 nm) was performed to fabricate the finger electrodes.

*Characterization*: XRD measurements were conducted by a Bruker D2 Phaser X‐ray diffractometer (XRD) with Cu K_α_ (*λ* = 0.154 nm) as the radiation source. Raman spectroscopy measurements were carried out by a Renishaw raman spectrometer at 514 nm. AFM measurements were performed with a Digital Instruments 3100 microscope under tapping mode.

*Sensing Measurements*: H_2_ (50 000 ppm) in N_2_ was used as a starting gas and it was diluted with N_2_ to the desired concentrations by using mass flow controllers. For the measurements, target gas was flowed with 200 sccm flow rate through a small glass chamber (10 cm^3^ volume), where the sensor device is mounted and the resistance was recorded by Keithley multimeter (2100). For ammonia sensing measurements, 50 ppm ammonia gas in N_2_ was used. For ethanol and acetone sensing measurements, the desired amount of liquid acetone and ethanol (calculated by using ideal gas law) were evaporated in a closed chamber, in which the concentrations of the solvents correspond to 50 000 ppm. For the recovery of the sensors, air was introduced into the chamber.

*Fabrication of FET Devices and Transport Measurements*: MoS_2_ nanosheets and MoS_2_ nanosheet‐PdCl_2_ dispersed in ethanol were spin coated on a SiO_2_ (300 nm thick) coated Si (high doped) substrate. A subsequent annealing process at 400 °C in forming gas environment was performed in order to reduce PdCl_2_. Source and drain electrodes were fabricated by photolithography and subsequent evaporation of Ti/Au (10/150 nm). Transport measurements were conducted by B1500 Agilent semiconductor device analyzer.

## Supporting information

As a service to our authors and readers, this journal provides supporting information supplied by the authors. Such materials are peer reviewed and may be re‐organized for online delivery, but are not copy‐edited or typeset. Technical support issues arising from supporting information (other than missing files) should be addressed to the authors.

Supplementary
