# Supplementary material for: MoS2 Nanosheet–Pd Nanoparticle Composite for Highly Sensitive Room Temperature Detection of Hydrogen
Source: Adv Sci (Weinh). 2015 Mar 2;2(4):1500004. doi: 10.1002/advs.201500004 (PMC5115349; doi:10.1002/advs.201500004)
Supplement: Supplementary file 1 — Supplementary [file ADVS-2-0j-s001.pdf]

Copyright WILEY-VCH Verlag GmbH & Co. KGaA, 69469 Weinheim, Germany, 2015.

ADVANCED SCIENCE
Open Access

Supporting Information

for *Adv. Sci.*, DOI: 10.1002/advs.201500004

MoS_2 Nanosheet–Pd Nanoparticle Composite for Highly Sensitive Room Temperature Detection of Hydrogen

*Cihan Kuru, Chulmin Choi, Alireza Kargar, Duyoung Choi, Young Jin Kim, Chin Hung Liu, Serdar Yavuz, and Sungho Jin**

Supporting Information

MoS_2 nanosheet-Pd nanoparticle composite for highly sensitive room temperature detection of hydrogen

*Cihan Kuru, Chulmin Choi, Alireza Kargar, Duyoung Choi, Young Jin Kim, Chin Hung Liu, Serdar Yavuz and Sungho Jin**

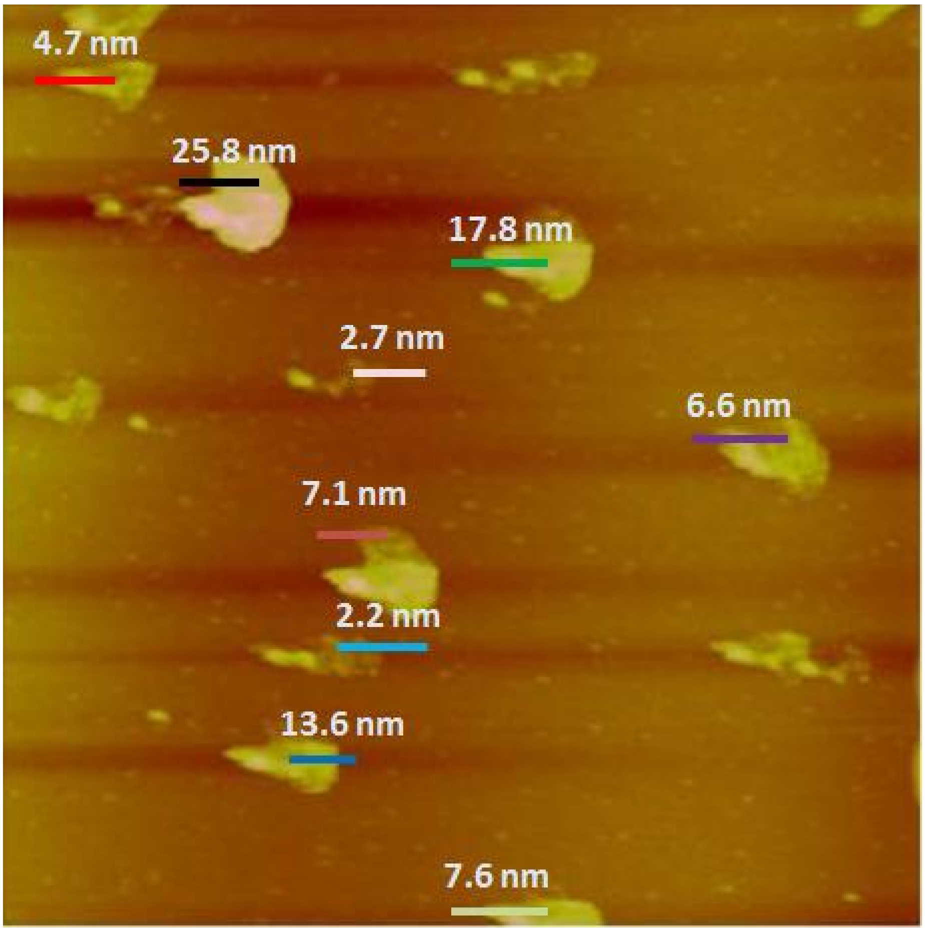

Figure S1. AFM image of MoS_2 flakes dispersed on a Si substrate.

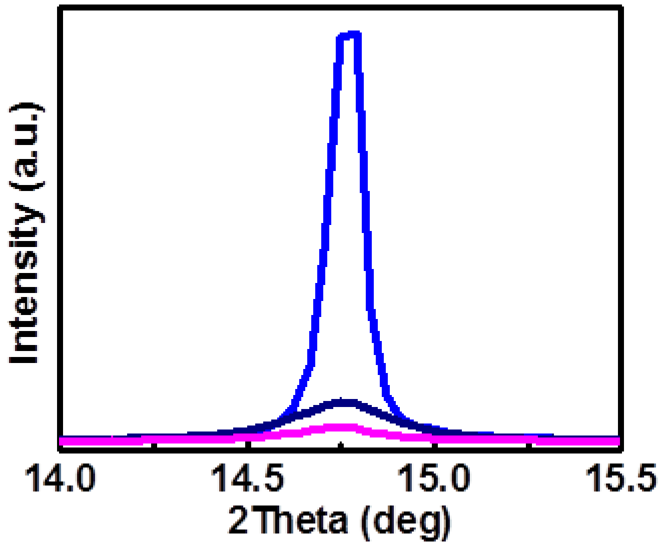

Figure S2. (002) XRD peak of bulk MoS_2 (blue curve), MoS_2 nanosheets (navy curve) and MoS_2-Pd composite (magenta curve).

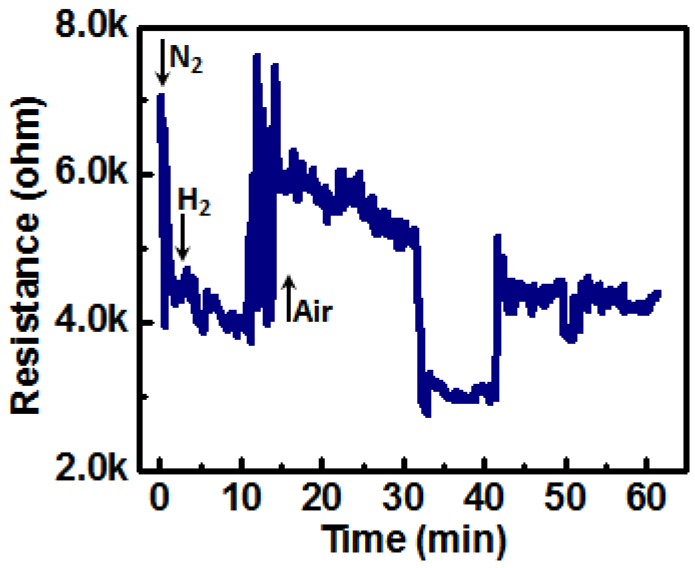

Figure S3. Electrical response of MoS_2 nanosheets to 50,000 ppm H_2.

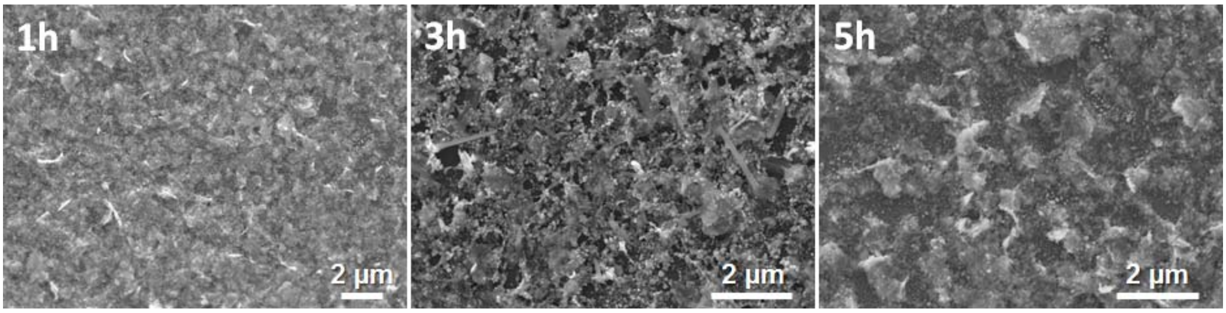

Figure S4. SEM images of MoS_2-Pd composites annealed at 500° C for (a) 1h, (b) 3h and (c) 5 h.

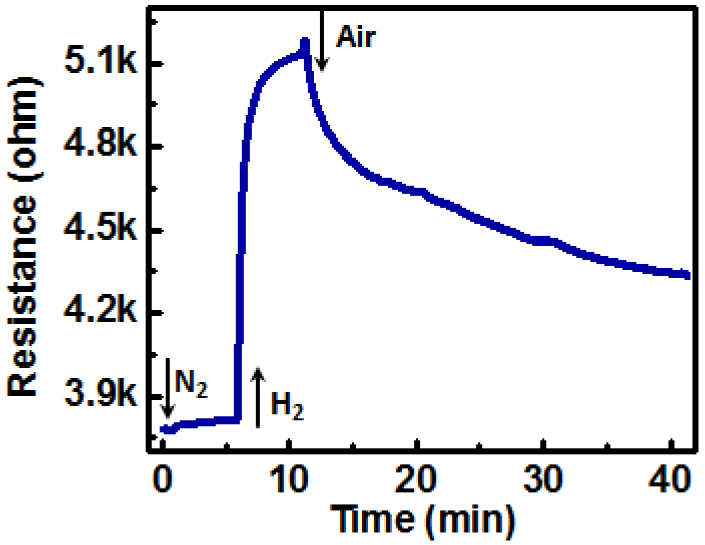

Figure S5. Electrical response of graphene-Pd composite to 50,000 ppm H_2.

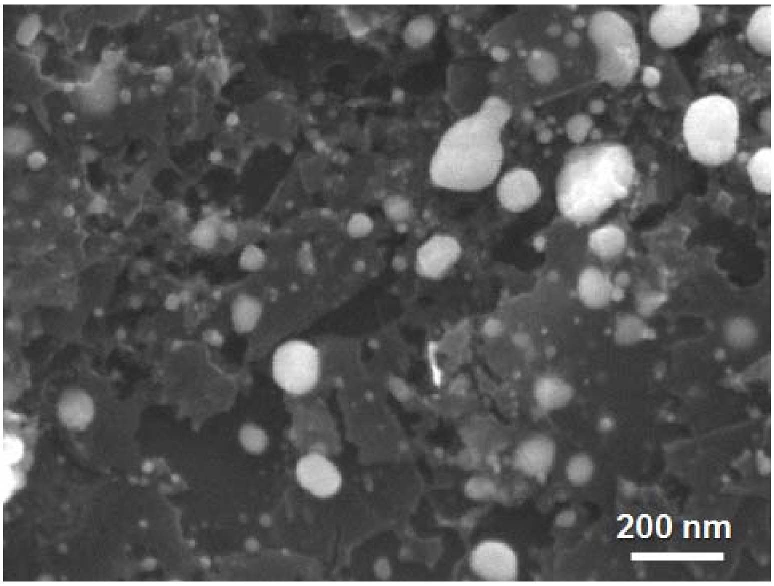

Figure S6. SEM image of graphene-Pd composite.
